# CT-derived vessel segmentation for analysis of post-radiation therapy changes in vasculature and perfusion

**DOI:** 10.3389/fphys.2022.1008526

**Published:** 2022-10-17

**Authors:** Antonia E. Wuschner, Mattison J. Flakus, Eric M. Wallat, Joseph M. Reinhardt, Dhanansayan Shanmuganayagam, Gary E Christensen, Sarah E. Gerard, John E. Bayouth

**Affiliations:** ^1^ Department of Medical Physics, University of Wisconsin, Madison, WI, United States; ^2^ Roy J. Carver Department of Biomedical Engineering, University of Iowa, Iowa, IA, United States; ^3^ Department of Animal Science, University of Wisconsin, Madison, WI, United States; ^4^ Department of Electrical and Computer Engineering, University of Iowa, Iowa, IA, United States; ^5^ Department of Radiation Oncology, University of Iowa, Iowa, IA, United States; ^6^ Department of Radiation Medicine, Oregon Health Sciences University, Portland, OR, United States

**Keywords:** lung perfusion, post-RT vascular change, pulmonary vasculature segmentation, radiation-induced damage, ct-derived perfusion

## Abstract

Vessel segmentation in the lung is an ongoing challenge. While many methods have been able to successfully identify vessels in normal, healthy, lungs, these methods struggle in the presence of abnormalities. Following radiotherapy, these methods tend to identify regions of radiographic change due to post-radiation therapytoxicities as vasculature falsely. By combining texture analysis and existing vasculature and masking techniques, we have developed a novel vasculature segmentation workflow that improves specificity in irradiated lung while preserving the sensitivity of detection in the rest of the lung. Furthermore, radiation dose has been shown to cause vascular injury as well as reduce pulmonary function post-RT. This work shows the improvements our novel vascular segmentation method provides relative to existing methods. Additionally, we use this workflow to show a dose dependent radiation-induced change in vasculature which is correlated with previously measured perfusion changes (*R* ^2^ = 0.72) in both directly irradiated and indirectly damaged regions of perfusion. These results present an opportunity to extend non-contrast CT-derived models of functional change following radiation therapy.

## 1 Introduction

Vascular segmentation is an ongoing challenge. Several groups have attempted to develop robust vasculature segmentation algorithms. Of these, most models rely on knowledge about the features of vessels such as intensity, curvature, tubularity, centerline, and smoothness but all methods thus far face their own challenges (Jerman et al., 2015) (Sato et al., 1998) (Krissian et al., 2000) (Aylward and Bullitt, 2002) (Zhang et al., 2010) (Lavi et al., 2004) (Metz et al., 2007) (Buelow et al., 2005) (Agam et al., 2005) (Medical Imaging 2008: Image Processing, 2008).

In lung images, there is a natural contrast seen on CT imaging due to the high density difference between vessels and the lung parenchyma. Segmenting vessels in the lungs specifically has been addressed by various groups (Agam et al., 2005) (Xiao et al., 2011) (Shikata et al., 2004) (Medical Imaging 2008: Image Processing, 2008). However, all these methods struggle to address the issue that other structures besides vessels can have similar Hounsfield Unit (HU) intensities such as tumor nodules or dense lesions resulting from fibrosis, mucous, etc. Particularly, in regions of damaged lung, previous work has indicated that the algorithm struggles to distinguish damaged lung from vessel (Rudyanto et al., 2014).

Texture analysis presents an opportunity to identify particular radiographic abnormalities caused by medical intervention e.g. radiation therapy. These methods are robust and can identify several different textures in lung parenchyma such as ground glass, honeycombing, emphysema, consolidated, bronchovascular, and ground glass reticular (Uppaluri et al., 1997). In this work we use these texture analysis methods to improve upon existing vascular segmentation methods to produce a workflow capable of identifying vasculature accurately in the presence of damaged or abnormal lung tissue. Furthermore, we use this workflow to show a dose dependent radiation-induced change in vasculature which correlated with measured perfusion changes in both directly irradiated and indirectly damaged regions.

## 2 Methods

### 2.1 Swine model subject description

Previous studies have used the Wisconsin Miniature Swine (WMS) and detail their unique characteristics that make them an ideal model as well as show strong correlations between WMS and human response (Wallat et al., 2021) (Reed et al., 2010) (Wuschner et al., 2021a). In this work, two groups of five WMS (ten total) each received CT imaging and RT treatment. The swine ranged from 70–100 kg and were 14.4 ± 1.7 months of age. These two groups will be referred to as group A and group B, with differences between the groups described in the current section. All WMS were sedated to eliminate motion artifacts and mechanically ventilated to a consistent tidal volume of 1 L and respiratory rate of 15 breaths per minute matching the average tidal volume and respiratory rate of human subjects in a prospective clinical trial studying functional avoidance in the lung (NCT02843568). The animal care practices and all experimental procedures were approved by the University of Wisconsin Institutional Animal Care and Use Committee (IACUC). The drugs and methods of anesthesia and euthanasia were approved in compliance with American Veterinary Medical Association (AVMA) guidelines for anesthesia and euthanasia of swine. Both committees assured that all procedures were in compliance with ARRIVE guidelines.

#### 2.1.1 Treatment schemes and imaging schedule

For all ten swine in groups A and B, CT images were acquired both pre- and 3 months post-RT. At each time point, subjects received a four-dimensional CT (4DCT) and dynamic contrast-enhanced perfusion scans. The dynamic perfusion scans were performed following the procedure detailed in Wuschner et al. (Wuschner et al., 2021a).

**FIGURE 1:**
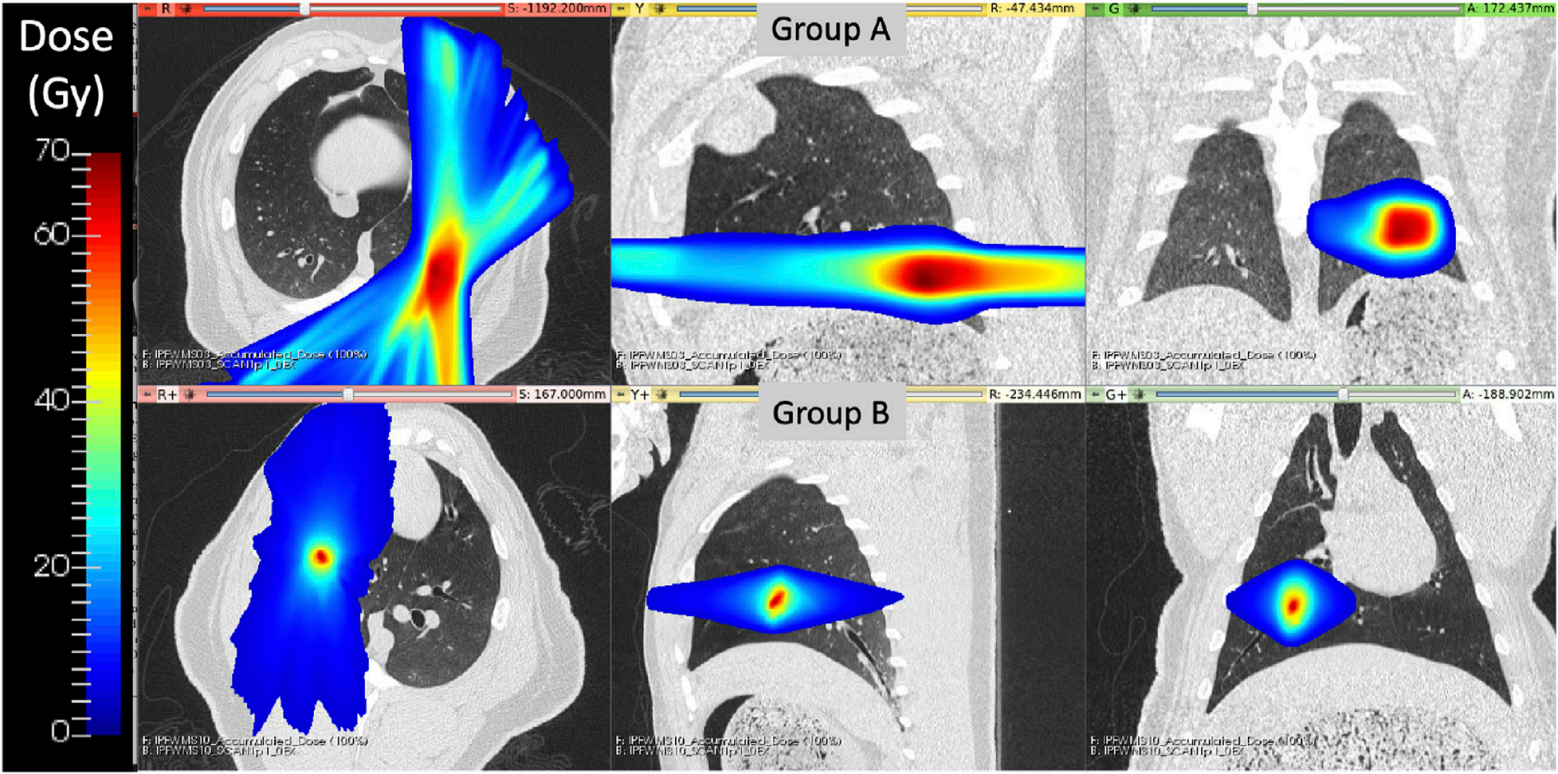
Delivered radiation dose distributions for WMS groups **(A)** and **(B)** show treatment differences. Group B had a more medial and cranial dose distribution with a smaller region of targeted high dose values.

All subjects underwent a five fraction stereotactic body RT (SBRT) course of 12 Gy per fraction totaling 60 Gy, however the two groups of swine were treated with two different forms of image-guided radiation therapy, as described below. The differences in delivery system were due to clinical availability at the time of the study. Figure 1 shows a representative dose distribution that was delivered to subjects in each group. For all subjects, the contralateral lung did not receive dose above 5 Gy.

In group A, the treatment planning target volume (PTV) was designated as the bifurcation of a vessel in the posterior base, near the left lateral chest wall, of the left lung. Treatment delivery was executed using an MRI-guided LINAC system (ViewRay, Cleveland Ohio) in order to maximize dose conformity and reduce the uncertainty of dose delivery due to respiratory motion. The ViewRay system continually monitors and gates treatment by acquiring 0.35 T MRI images and stopping treatment when the target is outside of the threshold view set.

For subjects in group B, the PTV was centered on a vessel and airway in the right upper lobe of the subject. This target location was selected to enable treatment response analysis to the directly irradiated vessels and distal pulmonary vasculature receiving moderate to low radiation doses. Treatments were delivered on the Radixact®linear accelerator with motion Synchrony treatment system (Accuray Incorporated, Sunnyvale, CA) in order to maximize dose conformity and reduce the uncertainty of dose delivery due to respiratory motion. Radixact®is a helical tomotherapy radiation therapy delivery system that contains an intrafraction motion management system called Synchrony^®^, which has been adapted from CK Synchrony (Schnarr et al., 2018). On the system, an x-ray tube and flat-panel kV imager are offset 90° from the megavoltage (MV) imager and beam. The kV imaging subsystem is used to periodically localize the target during treatment. For monitoring respiratory motion, light-emitting diodes (LEDs) were placed on the swines’ chest and identified with a camera mounted to the treatment table to provide the phase of respiration. The target can then be localized without implanted fiducials near the target using a motion correlation model. Further details of the model are described in Schnarr et al. (Schnarr et al., 2018).

For all swine, treatment fractions were delivered following a standard clinical SBRT schedule receiving each fraction with a day in between each delivery during weekdays and 2 days over the weekend. Subjects were mechanically ventilated to eight breaths per minute during treatments with inverted breathing to hold inhale longer than exhale.

### 2.2 Image analysis tools used

#### 2.2.1 Feature-learning vascular segmentation

In the 2012 VESSEL12 Grand Challenge run in conjunction with the IEEE International Symposium on Biomedical Imaging, several different approaches to vascular segmentation on non-contrast CT were proposed (Rudyanto et al., 2014). The top scoring approach by Kiros et al. utilized multi-scale patch-based feature-learning and implements the sparse coding principles described by Coates et al. (Coates and Ng, 2011) (Kiros et al., 2014). Since this method does not require joint learning, features are learned efficiently and quickly (Kiros et al., 2014). Since the conclusion of the challenge, Konopczynski et al. have improved upon the work of Kiros et al. by extending the method to learn 3D features in an unsupervised manner in a multi-scale scheme using dictionary learning *via* least angle regression (Konopczynski et al., 2016). Their method improved upon the accuracy achieved by Kiros et al. from 96.66 ± 1.10% to 97.24 ± 0.90% on the principle VESSEL12 data set (Konopczynski et al., 2016). The VESSEL12 challenge separately evaluated the ability of segmentation methods to distinguish several types of dense abnormalities from vessels. The datasets for these categories consisted of non-contrast CT images that contained vessels in the presence of dense lesions, which include atelectasis, fibrosis, and adhesive straining, as well as mucus-filled bronchi, which are airways that instead of being clear, are filled with liquid such as mucus (Rudyanto et al., 2014). Many of these are characteristic of the radiographic change that is seen in post-RT radiotherapy patients. In these categories, the methods of Konopczynski et al. achieved a sensitivity of 0.95, but struggled in specificity (achieved 0.13) meaning it classified several things as vessels that were not. This code is open-source and is code that was used in the “Vessel Segmentation” step shown in Figure 3 (Rudyanto et al., 2014). The output of this code is a probability map that indicates the probability of a given voxel containing a vessel.

#### 2.2.2 Texture analysis

To improve the specificity of the resulting vessel segmentation from the Konopczynski method described above, texture analysis was used to identify and remove false positives. The texture analysis method used is the Adaptive Multi-Feature Method (AMFM) developed by Uppaluri et al. at the University of Iowa (Uppaluri et al., 1997). This method classifies voxels of an input lung CT as one of seven textures: normal, ground glass, ground glass reticular, honeycombing, bronchvascular, emphysema, or consolidated.

The general procedure for performing the analysis is as follows. First, lung regions are identified on the CT scan using a multi-resolution convolutional neural network lung segmentaiton approach proposed by Gerard et al. (Gerard et al., 2020) (Gerard et al., 2021). Next, preprocessing was performed on the masked CT image using edgementation (Uppaluri et al., 1997). This method merges pixels in regions where the difference between the grey levels of adjacent pixels is small. From there, feature extraction is performed. The features extracted can be grouped into three categories; first order, second order, and the geometric fractal dimension. The first-order features were mean, variance, skewness, kurtosis, and grey-level entropy as described in Ferdeghini et al. (Ferdeghini et al., 1991). Eleven second order features were calculated. Five of these were derived from the run length matrix (short-run emphasis, long-run emphasis, grey-level non-uniformity, run-length non-uniformity, and run percentage) and the remaining six were derived from the co-occurrence matrix (angular second moment, entropy, inertia, contrast, correlation, and inverse difference moment) (Fleagle et al., 1994). The details of the geometric fractal dimension are detailed in Uppaluri et al. (Uppaluri et al., 1995). Each calculated feature is normalized for the size of the pixel and lung prior to optimal feature extraction. Optimal feature extraction is performed using the divergence method and correlation analysis with labeled training data that is classified as 1 of the seven textures by an experienced radiologist (Andrews and Swartzlander, 1973). Finally, classification is performed using a Bayesian classifier (Sonka et al., 1993).

**FIGURE 2:**
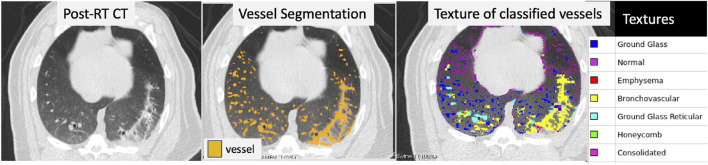
Example of a post-RT CT scan showing radiographic damage (left frame), the resulting vessel segmentation (middle frame), and the texture classification for each voxel (right frame) that was classified as a vessel. The vessel segmentation classifies several voxels in the area of the CT showing radiographic change as vessel that are likely false positives. Texture analysis was performed over the entire lung and is shown masked by the voxels that were identified as vessel in the third panel. The textures of the voxels in the region of false-positives are bronchovascular, consolidated, or ground-glass reticular which are expected radiation-induced textures.

Figure 2 shows the result of applying the AMFM texture analysis to a post-RT CT and masking it by voxels that were classified as vessels using the method described above. The vessel segmentation classifies several voxels in the area of the CT showing radiographic change as vessel that are likely false positives. Texture analysis shows that the voxels in this region are bronchovascular, consolidated, or ground-glass reticular which are expected radiation-induced textures. Therefore, by removing any voxels that are both classified as a vessel and classified by one of these textures, we can remove the false positives in the vessel segmentation.

However it can also be seen that voxels that are clearly vessels in the right lung are classified as ground glass reticular. This is because the AMFM technique is designed to classify lung parenchyma, not vasculature. When the vasculature is large enough, the features of the vasculature are similar to that of ground glass reticular and thus they are classified as such. This does not occur with the smaller vasculature that are small enough such that their normalized do not classify as one of the removed textures. To address this, a third image processing step is needed to add back in the larger vasculature.

#### 2.2.3 Lung and large vessel segmentation

**FIGURE 3:**
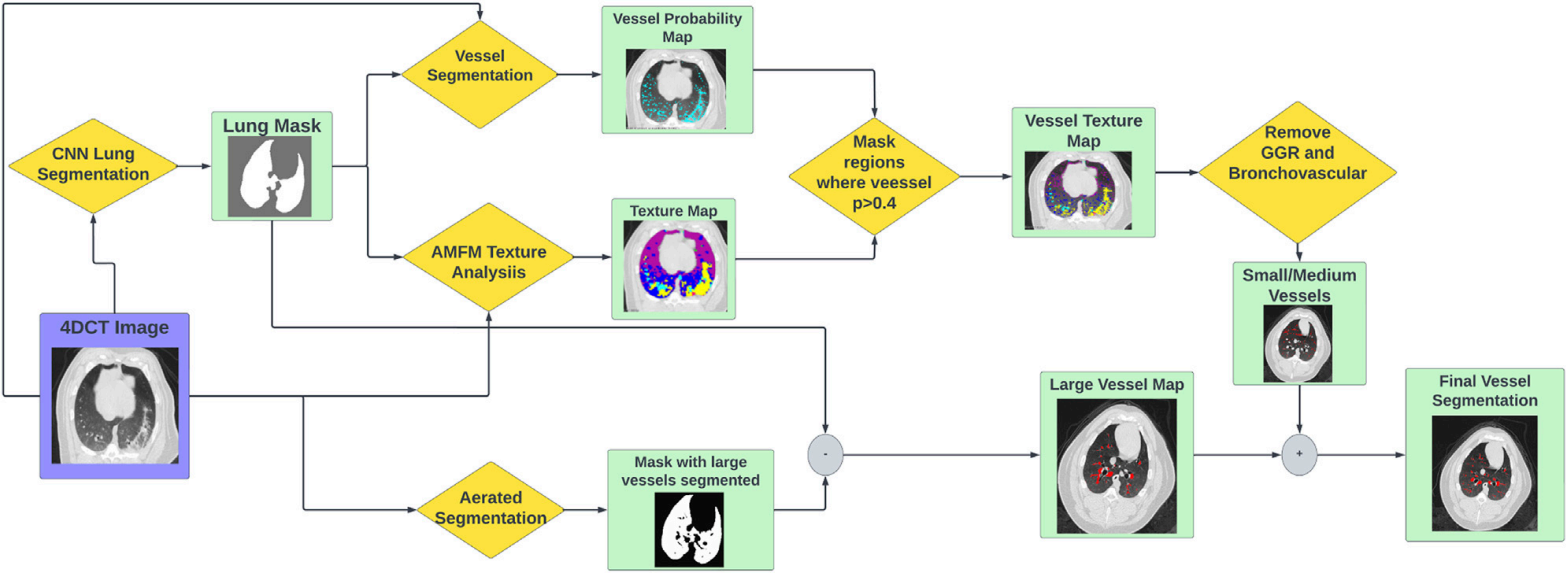
Flowchart showing the workflow to produce vascular segmentations using the tools described.

To add in the larger vasculature, two lung masking techniques are used. The first is a multi-resolution convolutional neural network proposed by Gerard et al.to perform lung segmentation (Gerard et al., 2020) (Gerard et al., 2021). The lung segmentation produced by this generates a smooth boundary at the mediastinum which includes the large vessels filled in (see Figure 3). The second method is an optimal thresholding method which is utilized to generate a mask of well-aerated regions. The aerated mask is subsequently smoothed and small holes are filled using morphological operations thus leaving the large vasculature unmasked. The difference image between the lung segmentation and the aerated segmentation is used to identify large vasculature (Gerard, 2018).

### 2.3 Vascular segmentation workflow

The full vascular segmentation workflow is detailed in Figure 3. The 4DCT image is input into the vascular segmentation, AMFM texture, CNN lung segmentation, and aerated segmentation codes. The AMFM texture map is masked by the vessel segmentation such that only vessel classified voxels remain (determined using a threshold of *p* > 0.4). Additionally, any voxels classified as vessel that are also identified as ground glass reticular or bronchovascular are removed. This produces the a map of the small and medium vasculature. Separately, the aerated segmentation is subtracted from the CNN lung segmentation to produce the large vasculature map. Finally, the large and small/medium vessel maps are added together to produce the final vessel segmentation.

### 2.4 Analysis of post-RT change

#### 2.4.1 Group A

All post-RT scans were deformably registered to the pre-RT scan using a B-spline registration algorithm (Cao et al., 2012) (Yin et al., 2009). The transformation matrix produced in the registration was then applied to the post-RT vessel segmentation to allow for voxel-wise comparisons. Analysis was performed in four dose bins; voxels receiving “no dose” ( < 5 Gy), “low dose” (5–20 Gy), “medium dose” (20–40 Gy), and “high dose” (above 40 Gy). In each dose bin, the volume of vasculature was calculated by summing the number of voxels classified as a vessel and multiplying by the voxel size. The percent change in vessel volume from pre to post-RT was then calculated using equation 1. Additionally student paired two-tailed t-tests were used to compare the pre and post-RT volumes of vasculature in each dose bin across the five swine.

$$\Delta VesselVolume\left(\%\right)=\frac{Volume_{\mathrm{post}}-Volume_{\mathrm{pre}}}{Volume_{\mathrm{pre}}}*100\%$$ (1)

#### 2.4.2 Group B

**FIGURE 4:**
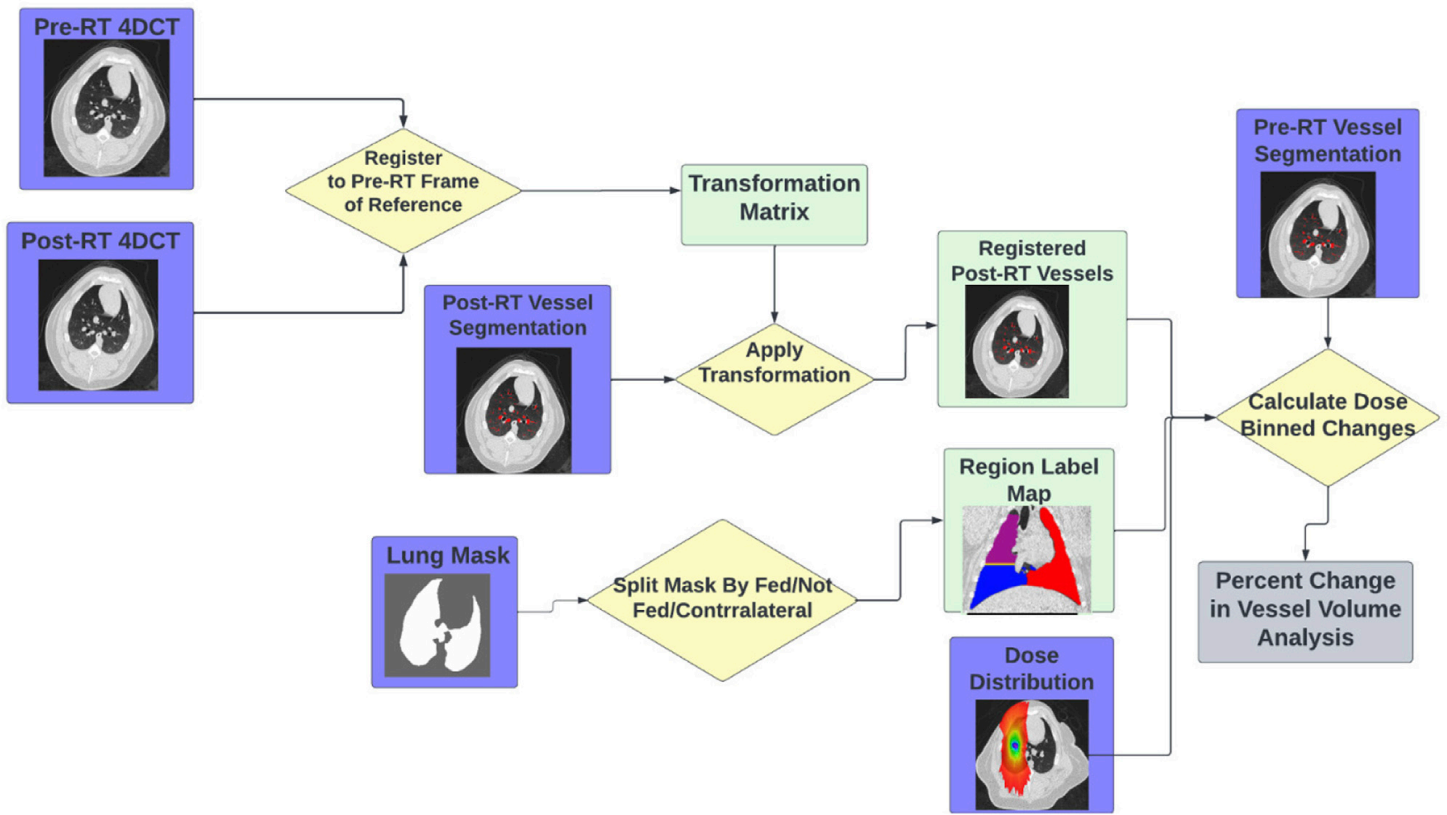
Flowchart showing the workflow to perform the indirect vascular change analysis using the tools described.

For the subjects in Group B, analysis was performed similarly to the subjects in Group A with the addition of analysis being split into direct and indirect change. The process for this analysis is summarized in Figure 4. Similarly to Group A, the pre and post-RT CTs are registered and the transformation matrix is used to bring the post-RT vessel map into the frame of reference of the pre-RT scan and dose distribution. However, in addition to masking by dose bin, the analysis is masked as being in either a “fed”, “not fed”, or “contralateral” region. Here “contralateral” refers to the left lung (left entirely unirradiated below 5 Gy), “fed” refers to regions that contain vasculature that branch from the vessel irradiated to the prescription dose, and “not fed” refers to regions that do not contain vasculature that branch from the vessel irradiated to the prescription dose. This results in 7 separate analysis regions (no, low, and medium dose in both the fed and not fed regions and the contralateral region). In each of these regions the percent change in volume of vasculature was calculated as described for group A using Eq. 1.

Additionally for these subjects, the perfusion change in each of the seven contours analyzed in the group B subjects was calculated from the contrast-CTs using the methodology described in Wuschner et al. (Wuschner et al., 2022).

## 3 Results

### 3.1 Improved vascular segmentation

**FIGURE 5:**
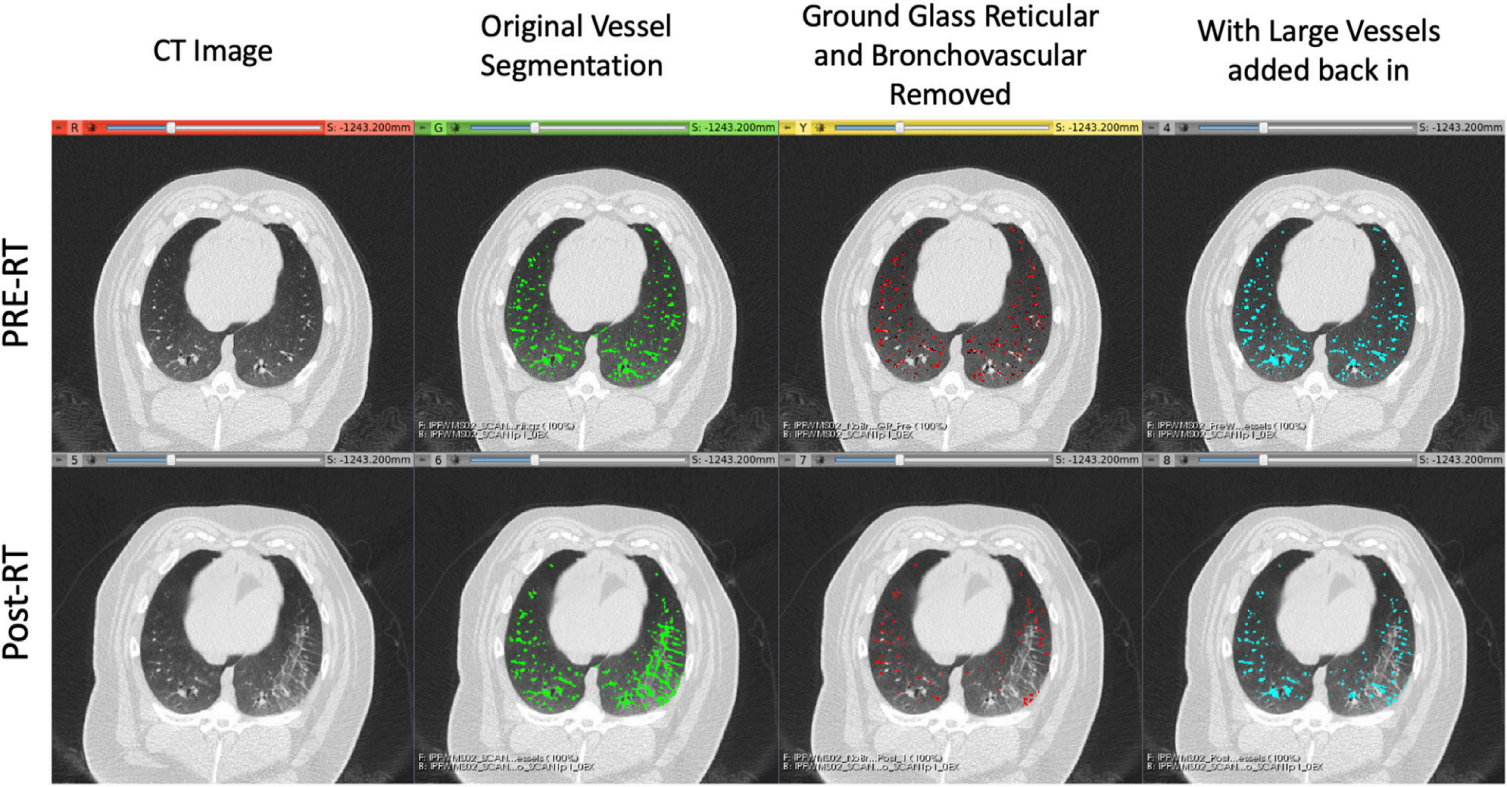
Example of the vessel segmentation algorithm results for a pre-RT (top row) and post-RT (bottom row) example. From left to right: the original CT, the vessel segmentation denoting voxels classified as vessels in green, the result of removing voxels that were classified as both vessels and either ground glass reticular or bronchovascular (red), and finally the result of adding back in the large vessels (blue).

**FIGURE 6:**
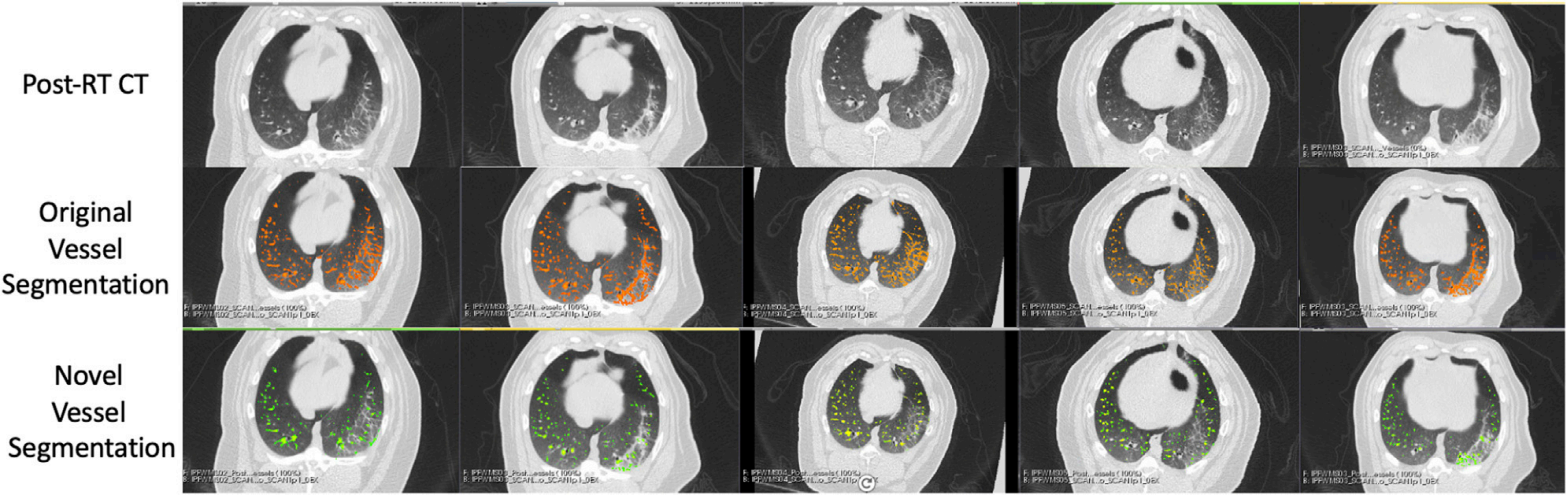
Segmentation results in the five Group **(A)** swine. Each column represents a subject where the top row shows the post-RT CT image in an axial slice showing the post-RT radiographic change. The middle row shows the original vessel segmentation overlayed on the CT in red which in all subjects classified damaged regions of the lung as vessel. The bottom row shows the result of the novel vessel segmentation workflow overlayed on the CT in green. In all subjects, the apparent quality of the vessel segmentation improves in the regions of radiographic change as indicated by the reduction in large connected regions being identified. The resulting segmentation appears to align with vessels that can be observed in the CT in both irradiated and non-irradiated regions.

**FIGURE 7:**
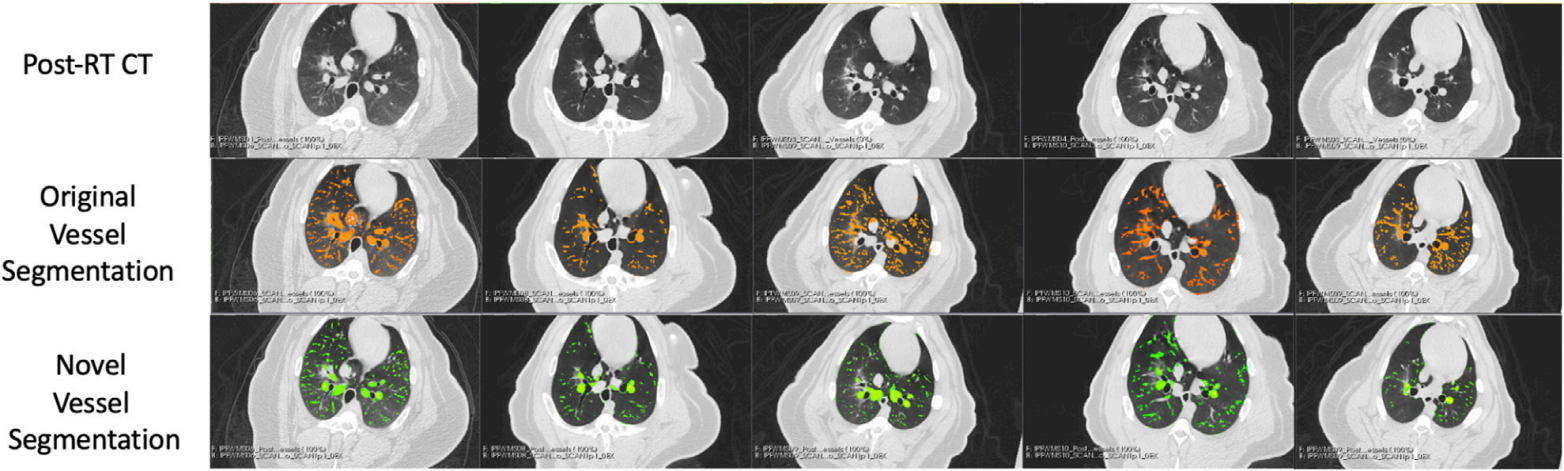
Segmentation results in the five Group **(B)** swine. Each column represents a subject where the top row shows the post-RT CT image in an axial slice showing the post-RT radiographic change. The middle row shows the original vessel segmentation overlayed on the CT in red which in all subjects classified damaged regions of the lung as vessel. The bottom row shows the result of the novel vessel segmentation workflow overlayed on the CT in green. In all subjects, the apparent quality of the vessel segmentation improves in the regions of radiographic change as indicated by the reduction in large connected regions being identified. The resulting segmentation appears to align with vessels that can be observed in the CT in both irradiated and non-irradiated regions.

**FIGURE 8:**
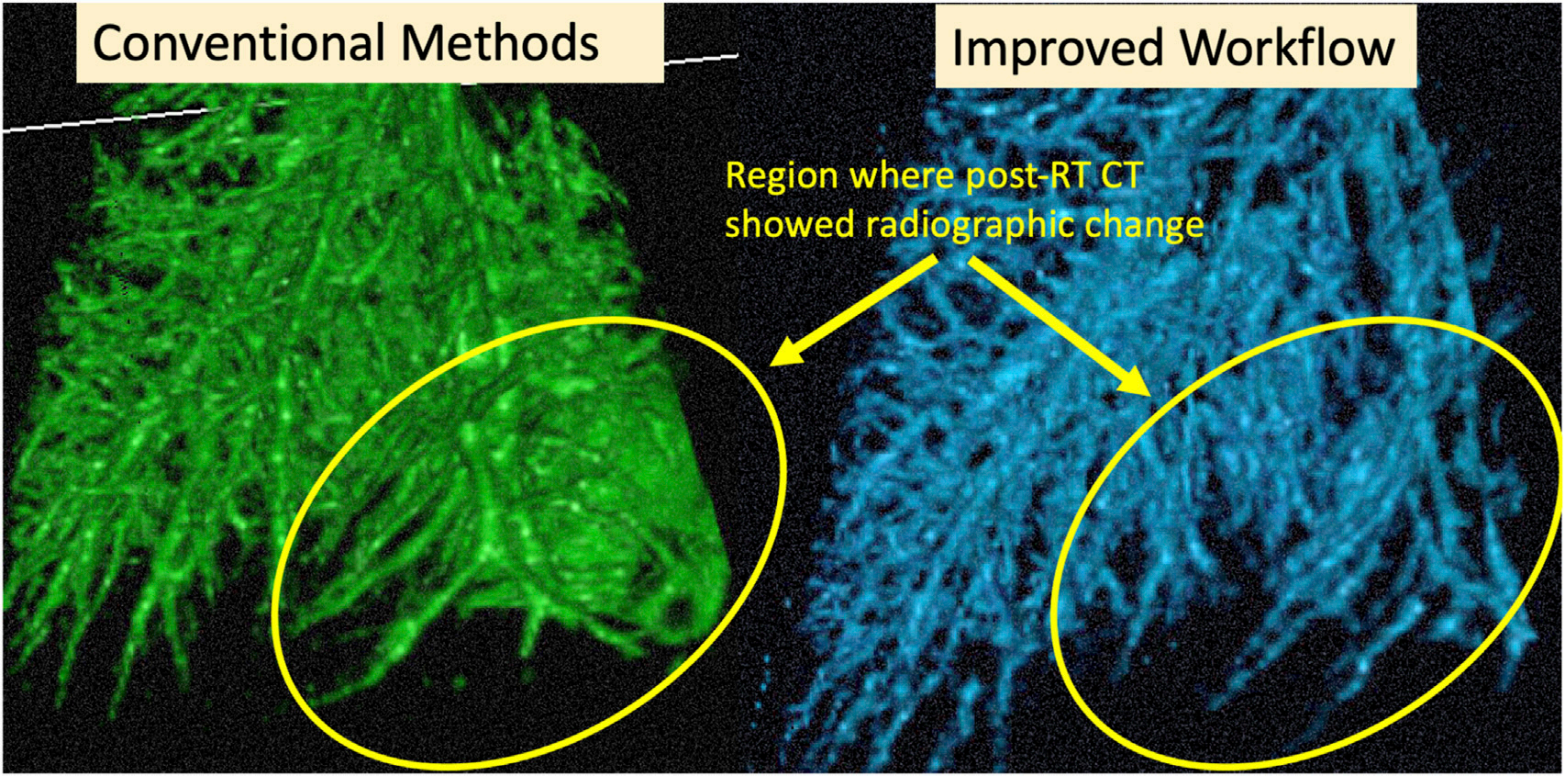
Example cropped 3D Rendering of an example subject. Circled regions show where the CT showed radiographic change post-RT. We see the result of the over-segmentation in the conventional method and the improvement on this using the proposed method.

An example of the vessel segmentation algorithm is shown in Figure 5. The post-RT original vessel segmentation classifies regions of radiographic change as vessel. When removing the ground glass reticular and bronchovascular voxels, in both the pre and post-RT examples it is observed that the larger vessels are no longer segmented. In the post-RT case it can also be seen that the radiographic change region is no longer classified as vessel. When large vasculature is added back in both pre and post-RT resultant vessel maps appear to be consistent with the vessels observed on the original CTs. Figures 6, 7 show the improvements made by the novel vessel segmentation in an axial slice of all of the subjects used in this work. In all subjects, it is clear that radiographic change is falsely classified as vessel in the original vessel segmentation method. This is further highlighted in Figure 8 where a side by side 3D rendering shows the effect of this false classification. The conventional method, in the regions denoted as having radiographic change, are so over-segmented that you cannot distinguish the true vasculature in this region and it just appears as a large condensed structure. However, when using the novel segmentation workflow presented in this work, we see that it appears specificity is improved in those regions of radiographic change while preserving the sensitivity of the segmentation in the rest of the lung. The improved workflow 3D rendering shows a connected vascular tree in these regions. In the group B swine, the result is more subtle but this is due to the fact that the radiographic change in these swine was not as drastic as the group A swine in a single axial slice. This is due to the differences in dose distribution delivered as well as differences in the size of vasculature irradiated.

### 3.2 Post-RT changes in vasculature

**FIGURE 9:**
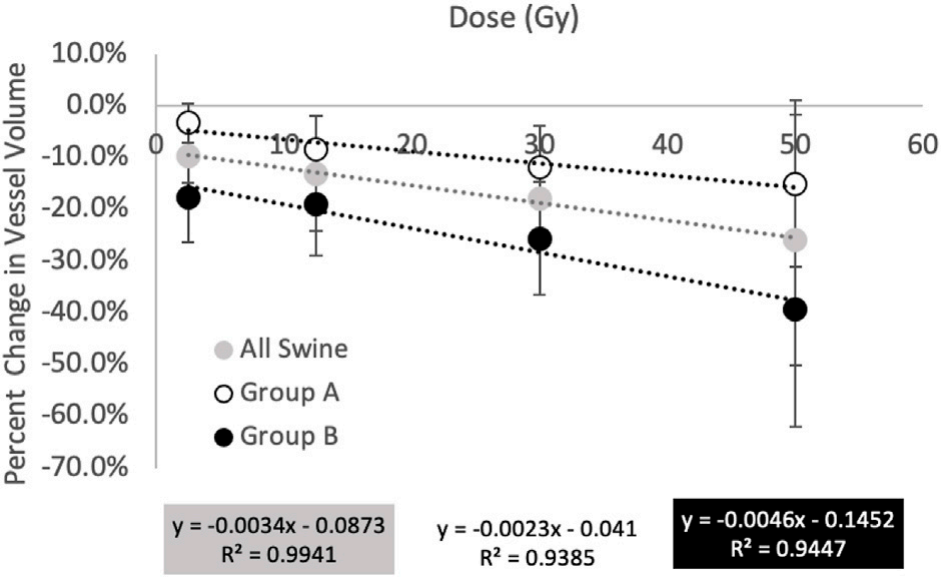
Summary of percent changes in vessel volume as a function of dose. All groups show increasing reductions in vessel volume with increasing dose however the magnitude of the changes differs in behavior between groups **(A)** and **(B)**. Group **(A)** shows minimal change in the unirradiated dose bin while Group **(B)** shows a large change.

Figure 9 and Table 1 show a summary of the percent changes in volume of vessel in each of the dose bins. For the group B subjects, analysis is not split into fed and not fed regions in this figure. Each point on the graph is plotted at the center of the dose bin it represents and is the average percent change of the five subjects analyzed (Group A or Group B) or 10 subjects analyzed (All Swine). All data sets show strong linear correlation with dose where the reduction in vascular volume increases with increasing dose. However, there is a difference in behavior between Group A and Group B where Group A shows minimal change in the unirradiated dose bin (-3.3 ± 3.8%) while Group B shows a large change (-17.8 ± 5.3%).

**TABLE 1:** Summary of percent changes in vessel volume. Values in table are entered as average (standard deviation) of the 10 swine (all swine) or of the five swine (Group A or Group B). Statistically significant values (*p* < 0.05) are denoted with a *.

**Dose (Gy)**	**All swine**	**Group a swine**	**Group B swine**
2.5	-9.7% (8.7%)*	-3.35% (3.8%)	-17.8% (5.3%)*
12.5	-13.1% (10%)	-8.4% (6.5%)*	-19.1% (11.1%)
30	-18% (10.9%)*	-11.8% (7.9%)	-25.7% (9.4%)*
50	-25.9% (22.7%)*	-15.1% (16.1%)	-39.4% (24.2%)*

**FIGURE 10:**
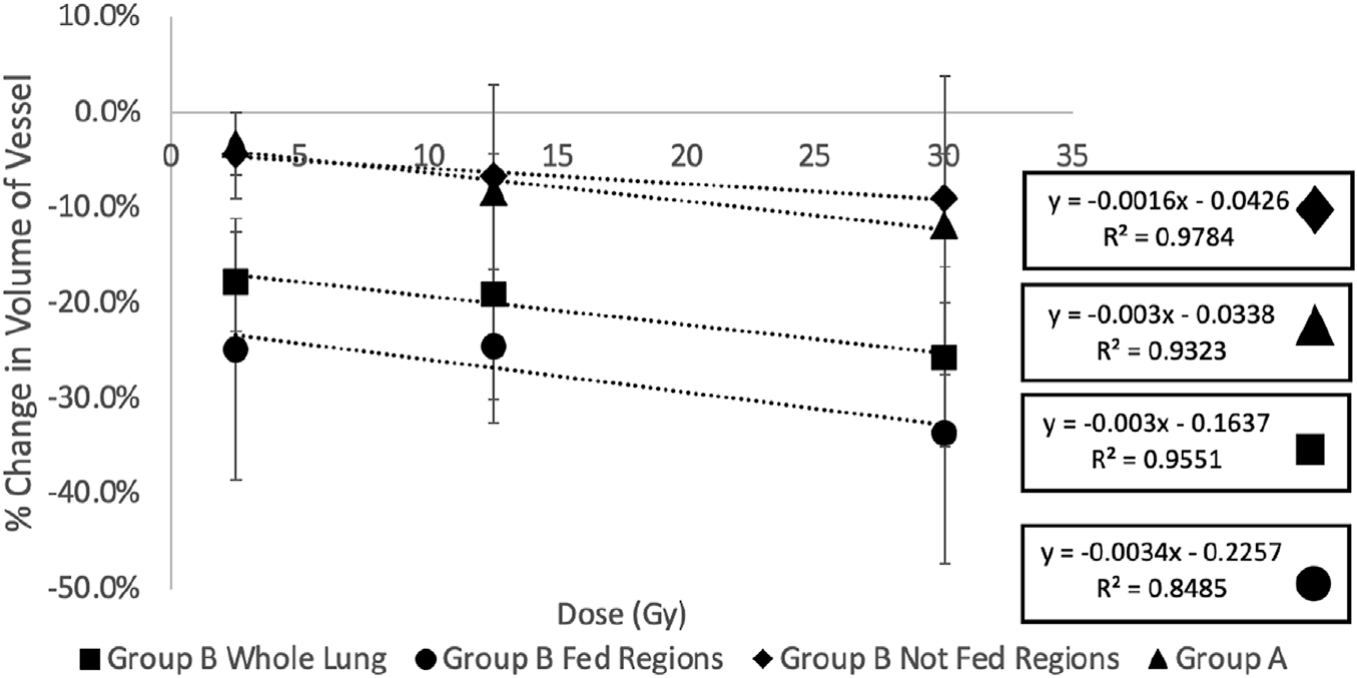
Summary of the percent changes in volume of vessel in each of the dose bins for the group **(A)** swine (all not fed), group **(B)** swine, and split group **(B)** swine results masked by being in a fed or not fed region. All data sets show strong linear correlation with dose where the reduction in vascular volume increases with increasing dose. The percent changes in the not fed regions are very similar to the group **(A)** percent changes while the fed regions show significantly higher magnitudes of change.

Figure 10 and Table 2 show a summary of the percent changes in volume of vessel in each of the dose bins with the additional group B analysis. All data sets still show strong linear correlation with dose where the reduction in vascular volume increases with increasing dose. The percent changes in the not fed regions are very similar to the group A percent changes while the fed regions show significantly higher magnitudes of change.

**TABLE 2:** Summary of percent changes in vessel volume. Values in table are entered as average (standard deviation) of the five swine in each group. Statistically significant values (*p* < 0.05) are denoted with a *.

**Dose (Gy)**	**Group a swine**	**Group B not fed regions**	**Group B fed regions**	**Group B whole lung**
2.5	-3.4% (3.8%)	-4.4% (3.3%)	-24.9% (13.7%)	-17.8% (5.3%)*
12.5	-8.4% (6.5%)*	-24.6% (8.1%)	-6.7% (11.2%)	-19.1% (11.1%)
30	-11.8% (7.9%)	-33.7% (13.7%)*	-9.1% (15.6%)	-25.7% (9.4%)*

### 3.3 Correlation of vasculature and perfusion change

**FIGURE 11:**
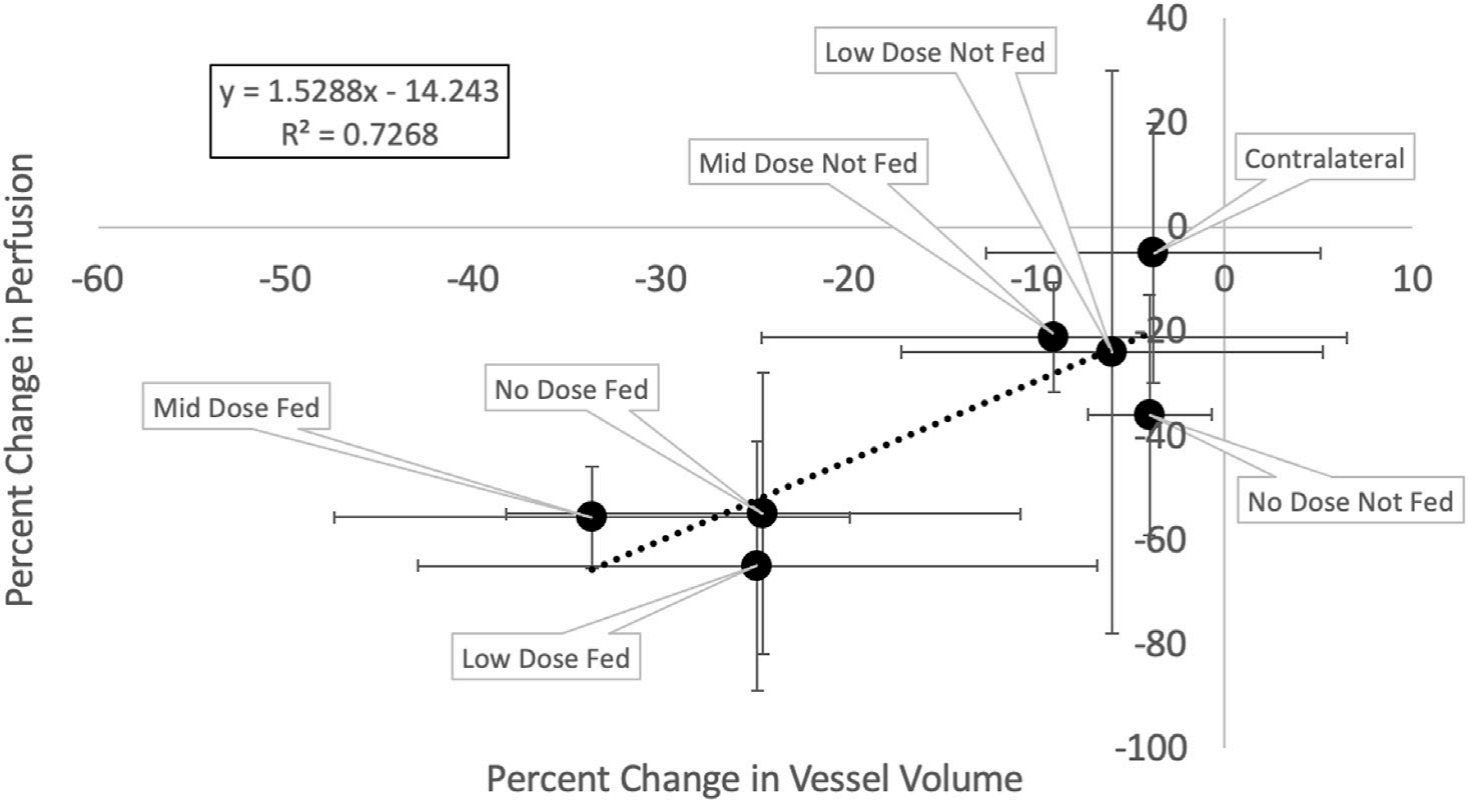
Change in vessel volume vs change in perfusion. Each point represents a different analysis contour and is the average of the five swine subjects with error bars representing the standard deviations in each metric.

Figure 11 and Table 3 show the relation between the percent change in vessel volume and the percent change in perfusion with each analysis contour labeled. The percent change in perfusion values were analyzed on the same subjects and same contours and were previously reported in Wuschner et al. (Wuschner et al., 2022). The perfusion study showed an indirect effect where the fed vessels, regardless of dose, experienced large, statistically significant compared to pre-RT, perfusion reductions. However, the not fed vessels did not experience statistically significant changes except in the mid dose vessels indicating that the perfusion reduction was dose dependent (in the case of not fed regions) but also dependent on location relative to highly irradiated regions (fed regions). The contralateral lung in the perfusion study experienced no statistically significant perfusion change. A line of best fit is drawn on Figure 11 and shows good correlation (*R* ^2^ = 0.726) between the perfusion study results from our previous study, and the vascular change results in this study.

**TABLE 3:** Summary of vessel volume percent change and perfusion percent change in each contour analyzed. Data in the table is the same as the data shown in Figure 11. Entries are written as the average (standard deviation) of the five subjects. Perfusion results are as reported in Wuschner et al. (Wuschner et al., 2022). Statistically significant values (*p* < 0.05) are denoted with a *.

**Contour**	**Vessel volume change**	**Perfusion change**
No Dose Fed	-24.6% (13.7%)	-55% (27%)*
0–5 Gy		
Low Dose Fed	-24.9% (18.1%)*	-65% (24%)*
5–20 Gy		
Mid Dose Fed	-33.7% (13.7%)*	-55.7% (9.7%)*
20–40 Gy		
No Dose Not Fed	-4% (3.3%)	-36 (23%)
0–5 Gy		
Low Dose Not Fed	-6% (11.2%)	-24% (54%)
5–20 Gy		
Mid Dose Not Fed	-9.1% (15.6%)	-21.2% (10.5%)*
20–40 Gy		
Contralateral	-3.8% (8.9%)	-5% (25%)

## 4 Discussion

### 4.1 Improvements of vessel segmentation method

It can be noted that the results presented in this section are all qualitative. While the qualitative results are convincing and encouraging, we recognize that future work should involve further validation of this method using a quantitative analysis. To our knowledge however, there is no publicly available labeled ground-truth data-set for vascular segmentation in the presence of radiation-induced radiographic change or other similar high density lung damage that could be confused as vessel using standard segmentation methods. The only available ground-truth we are aware of only labels vasculature in normal healthy lungs which will not test the novelty of our segmentation workflow.

With this acknowledged, Figure 5 shows a clear qualitative improvement in accuracy in the segmentation. In the post-RT scan there is clear radiographic change in the slice shown in the right lung. The original vessel segmentation shown in green identifies this radiographic change as vasculature. This misclassification was consistent across subjects and highlights the limitations to using the vessel segmentation method developed by Konopczynski et al. (Konopczynski et al., 2016) alone.

In Figure 2 another example of this mis-classification is shown in the middle pane where the regions denoted in orange pick up radiographic damage in the left lung. This figure further shows the texture correspondence in these regions using the AMFM texture analysis where it is clear that the dorsal regions of the left lung, where the radiographic change and false vessel classification is observed, is comprised of ground glass reticular and bronchovascular textures. The effect of removing vessels of this classification is shown in the third pane from the left in Figure 5. It appears through qualitative inspection that the specificity is improved in the region showing radiographic change but in both the pre and post-RT cases the sensitivity is reduced in the detection of large vessels. Finally, the final pane shows the result of the third step where large vessels are added back in. Here we see the apparent quality of the vessel segmentation improves in both irradiated and non-irradiated regions and is now consistent with the observable vessels on the CT image.

Particularly to note, is that in the pre-RT scan, the original and final segmentations appear identical. This confirms for us that the step of removing textures is only necessary to reduce the false positives in the case of abnormal radiographic features. In this work where the swine lungs were healthy at pre-RT this serves to remove radiation-induced damage, but in the case of human subjects who may have lungs with pre-existing disease, this could be extended for use in pre-RT scans as well.

### 4.2 Changes in vasculature

All results show a linear relation between increasing dose and decreasing vessel volume suggesting that radiation dose causes vascular volume reduction. Potential mechanisms of this include constriction and atrophy. Vascular atrophy has already been observed in the Group A swine and the direct effects of this atrophy on perfusion were reported previously (Wuschner et al., 2021a). Pathological analysis on these swine has confirmed a loss of structure in the vascular wall in addition to reporting additional mechanisms of constriction as well as confirming the intraparenchymal hemorrhage hypotheses reported in (Wuschner et al., 2021a), (Wuschner et al., 2022), and (Marks et al., 2003) where vascular leakage was observed as a result of radiation dose delivered (Wuschner et al., 2021b).

However, there are a few differences between analysis groups that suggest unique physiological characteristics. Figure 10 shows the difference between the fed and not fed regions that received the same dose. The “fed” region receiving the same dose as the corresponding “not fed” region showed significantly larger magnitudes of reduction in vessel volume. We believe this to be indication of an “indirect effect” where regions receiving no or minimal dose experience large functional declines. This effect has been observed previously in several studies (Wallat et al., 2021) (Vicente et al., 2020) (Thomas et al., 2019) (Farr et al., 2018).

Furthermore, the results of the “not fed” regions agree closely with the results of the group A swine who were irradiated in an inferior region of the lung. This means the region irradiated in the group A swine was centered on a small vessel that did not bifurcate multiple times to feed additional vasculature. These swine did not even have regions that were “fed” and received no dose and some did not have vessels irradiated that were “fed” and irradiated to low dose. These swine therefore, only experienced direct damage where the damage to the region is dependent primarily to the dose it received. This is also true of the “not fed” regions in the group B swine. However, the “fed” regions of the group A swine are also dependent on the dose to feeding vasculature; meaning if there is morphological change in the anatomy of a vessel that feeds the region, there will be downstream reduction in perfusion.

### 4.3 Correlation between vessel and perfusion change

Figure 11 shows a strong correlation exists between the observed reductions in vasculature and the observed reductions in perfusion (*R* ^2^ = 0.72). These results suggest that the change in vessel volume (a metric derived from a standard simulation 4DCT), is related to the change in perfusion to a region. Physiologically, this makes sense. Perfusion refers to the flow of blood through the capillary network surrounding the alveolar sacs. If the vascular tree is atrophied and blood cannot reach these capillaries, perfusion will reduce. Furthermore, the indirect effect will be magnified in the fed regions if blood leaks out of the vasculature several bifurcations prior to additional vasculature which supports why the percent changes in vessel volume are smaller than the percent changes in perfusion.

This combined with the dose dependency results shown in this work as well as our previous work (Wuschner et al., 2022) suggests that with enough subjects, a dose response model could be developed using the vascular tree as an input to predict the decline in perfusion to a region based on the dose it receives and the proximity of it to other locations receiving high dose on the vascular tree. This would allow for predictions in functional perfusion information to be made without the need for contrast.

This is a benefit for many reasons. The first benefit is for ease of integration into clinical workflow. Lung radiotherapy patients already receive a 4DCT in order to track lung motion and perform treatment planning. Previous perfusion studies have used methods such as SPECT or PET which require an additional scan and an injection of a radio-pharmaceutical (Ireland et al., 2007) (Farr et al., 2015a) (Thomas et al., 2019) (Marks et al., 2003) (Hopkins et al., 2012). The vascular maps can be derived from the same scans that are already used for treatment. While there are CT-derived perfusion scans, these all require the administration of iodine contrast. While being an additional step in clinical workflow, iodine can also be damaging to patient’s kidneys which is particularly important in the case of cancer patients who may already have compromised baseline renal function. Finally, these perfusion scans have limited field of view which limits the region of analysis and the number of vessels that can be analyzed. This leads to a large degree of variability in the perfusion derived measurements. This can be observed in Table 3 where the vessel change measurements have smaller standard deviations than the perfusion change measurements due to being able to analyze more vessels in the regions.

### 4.4 Comment on variability

There was a large degree of variability in these measurements as shown by the error bars on the plot in Figure 11 and those listed in Table 3. It is important to note that the sample size of this study was limited to only five subjects for this particular analysis since only the Group B swine were irradiated with enough distal vasculature and tissue to allow for this analysis. Notes on variations in response between subjects and potential causes for the large standard deviations in the perfusion work have previously been described by Wuschner et al. (Wuschner et al., 2022) and are applicable here as well. Future work should include extending this analysis to a larger pool of subjects to minimize the sensitivity of the average measurement to a single subject. Additionally, the new workflow, while it appears to yield a promising improvement, does still contain minor error which could contribute to some variability as well. Some of these errors can be visualized in Figure 5 where in the sub-pleural regions there are small regions segmented that do not look like vessels in the CT. This is likely due to errors in the aerated masking technique that struggles with damage that is so peripheral in the lung.

### 4.5 Application to functional avoidance radiation therapy

Lung cancer is one of the most commonly diagnosed cancers and is currently responsible for the highest percentage of cancer related deaths (American Cancer Society, 2022). A significant portion of these patients receive radiation therapy (RT) as part of their treatment depending on the stage of their cancer (American Cancer Society, 2022). However, many of these patients experience radiation-induced lung injuries as a result of treatment which decrease patient quality of life and can even be fatal (Marks et al., 2003).

Conventional methods use volumetric dose constraints to minimize toxicities, however these methods do not consider the local function of the lung which has been reported to be locally dependent and different by individual (Siva et al., 2015) (Farr et al., 2015b) (Faught et al., 2017) (Bates et al., 2009) (Vinogradskiy et al., 2013) (Yamamoto et al., 2011) (Shioyama et al., 2007).

Functional avoidance in RT treatment planning aims to do consider these personalized local dependencies by selectively avoiding high functioning regions of the lung. To do this, detailed dose response models are required. In recent years, multiple groups have begun developing these models and some have tested their efficacy in clinical trials (McDonald et al., 1995) (Mah and Dyk, 1988) (Mehta, 2005) (Graves et al., 2010) (Patton et al., 2018) (Koike et al., 2015) (Hopkins et al., 2012) (Zhang et al., 2010) (Vinogradskiy et al., 2013) (Wallat et al., 2020) (Wallat et al., 2021) (Bates et al., 2009) (Hoover et al., 2014) (Ireland et al., 2016) (Vicente et al., 2020). To date, the only prospective clinical trials using these techniques have been ventilation-based (Bayouth et al., 2019) and all non-contrast CT-derived methods have been exclusively ventilation based (Patton et al., 2018) (Vinogradskiy et al., 2013) (Vicente et al., 2020) (Castillo et al., 2021). This does not create a comprehensive model to accurately model the function regions that need avoidance. Perfusion based trials have been performed, however they have all been retrospective and utilized scans outside of normal clinical workflow which poses challenges as described previously (Ireland et al., 2007) (Siva et al., 2015) (Farr et al., 2015a) (Thomas et al., 2019). Having a bio-marker that can derive perfusion information from the same standard of care CT would allow for a more comprehensive model of function to be developed and tested in prospective clinical trials. Future work should involve performing these measurements on a large cohort of subjects to build a model then testing that model in a prospective clinical trial.

## 5 Conclusion

In this work we present a novel vascular segmentation workflow that shows significant observable improvements. Through qualitative inspection, it appears there is an improvement in accuracy in the presence of damage or abnormal radiographic features on a CT. Additionally we use this method to demonstrate a strong dose-response relationship on the morphology of segmented vasculature post-RT. Finally, we show that these measurements correlate with previously reported perfusion changes in the same subject cohort which presents an opportunity for this method to be a non-contrast CT-derived bio-marker for functional perfusion change. While future work should fully validate the method proposed *via* quantitative analysis, this work presents numerous potential benefits towards the advancement of functional avoidance treatment planning.

## Data Availability

The original contributions presented in the study are included in the article/supplementary material, further inquiries can be directed to the corresponding author.
